# Association of Infant Feeding Indicators and Infant Feeding Practices with Coexisting Forms of Malnutrition in Children under Six Months of Age

**DOI:** 10.3390/nu14204242

**Published:** 2022-10-12

**Authors:** Asif Khaliq, Darren Wraith, Yvette Miller, Smita Nambiar

**Affiliations:** 1 School of Public Health and Social Work, Queensland University of Technology, Brisbane 4059, Australia; 2 School of Nutrition and Dietetics, Queensland University of Technology, Brisbane 4059, Australia

**Keywords:** malnutrition, association, feeding, practice, infants, Pakistan

## Abstract

Breastmilk is the only recommended source of nutrition for infants below six months of age. However, a significant proportion of children are either on supplemental breastfeeding (SBF) or weaned due to the early introduction of solid/semi-solid/soft food and liquids (SSF) before six months of age. There is good evidence that exclusive breastfeeding (EBF) in infants below six months of age protects them from preventable illnesses, including malnutrition. The relationship between infant feeding practices and coexisting forms of malnutrition (CFMs) has not yet been explored. This study examined the association of different feeding indicators (continuation of breastfeeding, predominant feeding, and SSF) and feeding practices (EBF, SBF, and complete weaning) with CFM in infants aged below six months in Pakistan. National and regional datasets for Pakistan from the last ten years were retrieved from the Demographic Health Surveys (DHS) and UNICEF data repositories. In Pakistan, 34.5% of infants have some form of malnutrition. Among malnourished infants, 44.7% (~15.4% of the total sample) had a CFM. Continuation of breastfeeding was observed in more than 85% of infants, but less than a quarter were on EBF, and the rest were either SBF (65.4%) or weaned infants (13.7%). Compared to EBF, complete weaning increased the odds of coexistence of underweight with wasting, and underweight with both wasting and stunting by 1.96 (1.12–3.47) and 2.25 (1.16–4.36), respectively. Overall, breastfed children had lower odds of various forms of CFM (compared to non-breastfed), except for the coexistence of stunting with overweight/obesity. Continuation of any breastfeeding protected infants in Pakistan from various types of CFM during the first six months of life.

## 1. Introduction

### 1.1. Background

Breastmilk is a natural source of nutrition for newborns and infants [1]. It contains all nutrients that are essential for the growth and nourishment of a newborn and a growing infant, such as carbohydrates, proteins, fats, vitamins, and trace elements [2,3]. Breastmilk is enriched with all five immunoglobulins (Ig), such as IgA, IgD, IgE, IgG, and IgM, which provide immunity against various infections and common preventable diseases such as diarrhea, pneumonia, necrotizing enterocolitis, otitis media, neonatal septicemia, and various other preventable illnesses [1,2,3,4]. Benefits of receiving breastmilk continue over the life course, protecting from several non-communicable and metabolic diseases in adulthood, including diabetes and obesity [5]. Breastfeeding also protects nursing mothers from breast cancer, ovarian cancer, coronary heart disease (CHD), diabetes, and unplanned pregnancies [1,5]. The World Health Organization (WHO) and the American Academy of Pediatrics (AAP) recommend exclusive breastfeeding (EBF) for infants up to six months of age, and continued breastfeeding to two years and beyond if the dyad can continue [6,7].

Despite the universal recommendation and promising health outcomes associated with EBF, more than half of infants worldwide do not receive EBF for the first six months of their life [8]. Various medical and nonmedical reasons are responsible for an early cessation of EBF before the recommended age of six months, such as maternal illness, child illness, medication use by mother and/or child, maternal employment, subsequent pregnancy, and use of a pacifier [9]. Many studies supported that an insufficient supply of milk is the most common reason for an early cessation of EBF [9,10,11]. Supplemental breastfeeding (SBF) and complete weaning are two alternative practices to EBF adopted by mothers and/or caregivers [6,12,13]. SBF refers to the use of either water, formula milk, cattle milk, and solid/semi-solid foods together with breastmilk for feeding a neonate and infant below six months of age [6,13], while complete weaning refers to feeding solid, semi-solid/soft, and liquid foods after complete cessation of breastmilk before the first six months of a child’s life [14]. Both SBF and early initiation of weaning practices are associated with various types of standalone forms of malnutrition, such as wasting, stunting, underweight, and overweight/obesity [12,14,15].

CFM represents the simultaneous occurrence of two or more forms of nutritional disorders in an individual [16,17] (e.g., an individual who is both stunted and overweight/obese or underweight with wasting and/or stunting and/or both). In general, women of reproductive age and children below five years of age are highly vulnerable to various forms of nutritional disorders, including CFM [18]. Worldwide, more than two-thirds of malnourished children aged below five years reside in most of the South Asian and Sub-Saharan African countries [19,20]. Among South Asian countries, Pakistan is the second largest South Asian country, where the burden of malnutrition has been stagnant for the last four decades [18,19]. This stagnancy in the prevalence of pediatric malnutrition is associated with various preventable illnesses, including malnutrition, and malnutrition itself contributes to around half of deaths in children [21,22,23]. Previous studies have shown that an adherence to infant feeding effectively reduces the burden of neonatal and infant deaths owing to various preventable illnesses, including malnutrition, by 20% [24,25].

The evidence regarding the importance of breastmilk has been supported by various observational and experimental studies. Previous studies have provided good evidence for EBF and reduced risk of malnutrition in infants, but the relationship of EBF with various forms of CFM has not been explored. To the best of our knowledge, this is the first study to examine the relationship between different types of feeding indicators (continuation of breastfeeding (CBF), predominant feeding (PF), introduction of solid, semi-solid, soft, and liquid foods (SSF)) and feeding practices (EBF, SBF, and complete weaning practices) and various forms of CFM. Therefore, this study examined the association of different feeding indicators (CBF, PF, and SSF) and feeding practices (EBF, SBF, and complete weaning practices) with different forms of CFM in children aged 0 to 5.9 months using datasets from Pakistan Demographic and Health Surveys (PDHSs) and Multiple Indicator Cluster Surveys (MICSs).

### 1.2. Conceptual Framework

**Figure 1:**
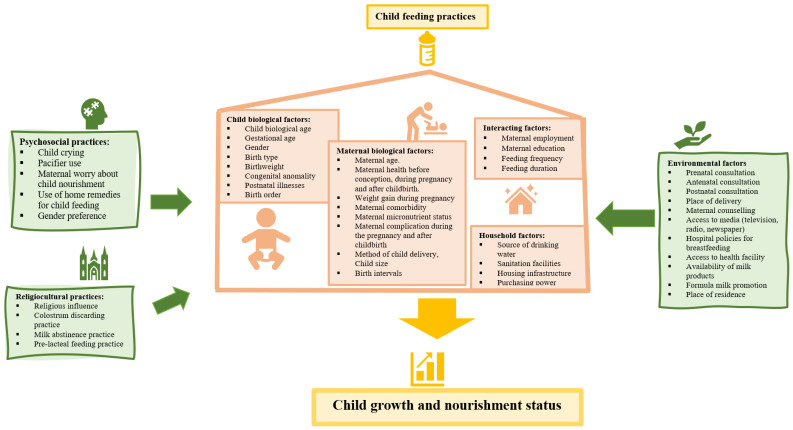
Conceptual framework indicating the relationship of an infant’s feeding practices with nutritional status.

The conceptual framework for assessing the relationship of infant feeding practices with their nutritional status is presented in Figure 1. At a microlevel, the nutritional status of a child is influenced by the child’s own biology (e.g., child biological age, gestational age, gender, birth type, birthweight, congenital anomality, and postnatal health/disease status), maternal biology (e.g., maternal age; maternal health before conception, during pregnancy, and after childbirth; weight gain during pregnancy; maternal co-morbidity; maternal micronutrient status; maternal complication during pregnancy and after childbirth; method of childbirth; child size; birth intervals), and interacting factors (e.g., maternal education, maternal employment status, feeding frequency, and feeding duration). However, at a macro level, several environmental, cultural, household, and psychological factors affect the feeding practices and nutritional status of a child (Figure 1).

## 2. Methodology

### 2.1. Datasets

This is a secondary data analysis of Pakistan Demographic and Health Survey (PDHS) and Multiple Indicator Cluster Survey (MICS) datasets, which were retrieved from the DHS program and from UNICEF, respectively, after formal registration and approval. The PDHS collects information relating to demography and health indicators using different sets of questionnaires at the national level. The MICS collects health and demographic data from children and their mothers at the regional level [26,27,28]. In this study, a total of ten different datasets, two from the last two PDHSs and eight from the MICS, were merged for assessing the relationship between CFM and different feeding practices that may be encountered in infants aged below six months. Data from the PDHS 1990–1991, PDHS 2006–2007, and MICS 1995 were excluded because they did not include most of the feeding indicators related to infant and young child feeding practices (IYCF) indicators.

### 2.2. Study Population, Sample Size, and Sampling Method

The target population in each DHS and MICS survey was women of reproductive age, who were interviewed using a multistage stratified cluster sampling method. Further detail about the sampling method has been presented elsewhere [17]. From the main sample of the study, data for infants aged below 5.9 months were analyzed, consistent with EBF guidelines proposed for infant and young child feeding (IYCF) [6]. Children were excluded if they were aged over 5.9 months, had missing anthropometry, or had anthropometric red flags (outliers). The World Health Organization (WHO) has described different ranges of anthropometric outliers for each anthropometric index. A cutoff value exceeding ±6.00 S.D. for length/height for age (LAZ/HAZ), ±5.00 S.D. for weight for length/height (WHZ), and of −6.00 and +5.00 S.D. for weight for age (WAZ) was considered an outlier [29,30]. After excluding data for all ineligible cases, we analyzed the data of 17,782 children (Supplementary Table S1).

### 2.3. Measurement of Outcome Variables

A series of steps were used for classifying the type of CFM. Firstly, data for all the children with a missing value for age and/or sex, weight, height/length, or measurement position were removed. Second, the anthropometric data were imported into the WHO AnthroCal^®^ version 1.6 for z-score calculation. WHO AnthroCal calculates four anthropometric indices: WHZ, WAZ, LAZ/HAZ, and body mass index z-scores (BAZ) for assessing the nutritional status of a child. In this study, WHZ, WAZ, and HAZ were considered for assessing various forms of malnutrition, while BAZ was excluded because it is a poor predictor for assessing nutritional status in young children [31,32]. Thirdly, all anthropometric outliers (outlined in Section 2.2) were removed from the analysis files. Finally, the nutritional status of each child across nine mutually exclusive categories was determined, of which four represented CFM (e.g., coexistence of: underweight with wasting; underweight with stunting; underweight with both wasting and stunting; and stunting with overweight/obesity), four represented standalone forms of malnutrition (e.g., wasting, stunting, underweight, and overweight/obesity), and one represented healthy nutritional status. Further details regarding the assessment of nutritional status in this research are reported elsewhere [17].

### 2.4. Measurement of Independent Variables

In each PDHS and MICS survey, data related to feeding indicator and feeding practices were obtained from mothers using a food list proposed in the *Infant and young child feeding* (*IYCF*) guidelines given by the World Health Organization (WHO) and UNICEF for children aged below two years. Parents of children below two years responded either yes or no to each food item consumed by their children in the last twenty-four hours. The responses to different food items were then used to derive a set of feeding indicators. In other words, the feeding indicators reflect the consumption of specific types of food by an individual, such as the use of breast milk in children below 6 months, the use of iron and folate supplementation in children below 6 months, and the use of solid food in children below 6 months. The current IYCF guidelines for 2021 have set 17 feeding indicators (there were 15 indicators in the 2010 IYCF guidelines) for improving the health and nourishment of children below two years of age, some of which are age-specific [33,34]. We examined the relationship of three infant feeding indicators with various forms of CFM: *continuation of breastfeeding* (*BF*), *predominant feeding* (*PF*), *and solid, semi-solid and liquid feeding* (*SSF*). Continuation of BF can be defined as consumption of breastmilk by an infant in the last 24 h. Any neonate or infant who consumed water, and/or juice, and/or clear broth, and/or clear tea without milk in addition to breastmilk were categorized as PF. Infants who consumed animal milk and/or formula milk, yogurt, porridge, tea with milk, or soft and semi-solid liquid and food were categorized as SSF.

The relationship of various forms of CFM with infant feeding practices was also investigated, which indicate the overall dietary consumption within the 24 h window period. In general, the feeding practices denote a combination of feeding outcomes derived from the feeding indicators. Three different types of feeding practice (EBF, SBF, and complete weaning) were derived following IYCF guidelines using a number of questions related to feeding indicators [33,34] (Supplementary Table S2).

An exclusively breastfed child was one who consumed breastmilk either alone or together with ORS or multivitamins/minerals a day before data collection.

A supplementary breastfed child was one who consumed solid food or semi-solid food or liquid diet or formula milk or predominant feeding together with breastfeeding. Based on the consumption of different types of foods and liquids, four different types of SBF practices were derived: (a) coadministration of breastmilk with infant formula, (b) coadministration of breastmilk with animal milk, (c) coadministration of breastmilk with water, juice, broth, and other liquids, and (d) coadministration of breastmilk with solid, semi-solid, and soft food.

A weaned child was one who consumed solid food, semi-solid food, a liquid diet, formula milk, or predominant feeding, either alone or in combination, before 6 months of age in the absence of breastfeeding.

### 2.5. Covariates

Several covariates were identified that could potentially influence the relationship between feeding practices and nutritional status of infants under 6 months of age. We considered maternal, child, household, environmental, cultural, and psychosocial factors for assessing the relationship of infant feeding practices with their nutritional status. In this study, some covariates were not available in the DHS and MICS datasets. Covariates considered for the analysis included:

**Child factors:** biological age (0 to 1.9 months, 2 to 3.9 months, and 4 to 5.9 months), sex (male or female), and postnatal illnesses (yes or no).

**Maternal factors:** maternal education, categorized as none, primary education, and secondary or higher education.

**Household factors:** socioeconomic status, which was pre-calculated in each dataset with five categories: poorest, poorer, middle, richer, and richest. Place of residence was in two categories: urban and rural

### 2.6. Data Management and Data Analysis

Different statistical software packages (Microsoft Excel version 2019, SPSS Version 27, and Jamovi Version 2.3.17) were used to analyze data. Before performing inferential statistics, four data files, each representing a type of CFM with its corresponding reference category, were created. The reference category for coexistence of underweight with wasting, stunting, and both was “underweight”, while the reference category for the coexistence of stunting with overweight/obesity was “stunting”. Data from each new file was then used for inferential analysis.

In this study, the inferential analysis was performed at three levels. Firstly, the association of each feeding indicator: continuation of breastmilk, predominant feeding, and solid, semi-solid, and soft foods was measured with each form of CFM. Secondly, the association of each feeding practice, such as EBF, SBF, and early weaning practices and CFM were examined. Lastly, association of each type of SBF was assessed with different forms of CFM. During the inferential analysis, at first, the unadjusted odds for each study outcome using binomial regression were calculated (Supplementary Table S4). A 95% confidence interval was used to indicate the uncertainty of the estimates or results. In preliminary analysis, we did not find a high degree of collinearity between any covariates; thus, all covariates were considered for calculating the adjusted odds of each study outcome.

### 2.7. Ethical Clearance

The data of this study were retrieved formally from the DHS and UNICEF data repositories. Ethical clearance was obtained from the University Human Research Ethics Committee (UHREC) of Queensland University of Technology, Brisbane, Australia (Approval number 2000000177).

## 3. Results

### 3.1. Health, Demographic, and Feeding Profile of the Study Sample

A total of 17,782 infants aged between 0 and 5.9 months were analyzed in this study. A description of the study sample is presented in Table 1. The prevalence of common preventable illness was 33.6%. Over a third of infants aged below six months had malnutrition, and among malnourished infants, 44.7% (~15.4% of total population) had CFM.

More than two-thirds of children with CFM had either a coexistence of underweight with wasting or coexistence of underweight with stunting. The prevalence of coexistence of stunting with overweight/obesity in infants under six months was 14.6%.

Continuation of breastfeeding in children aged below six months was observed in more than 85% of infants. Early initiation of solid, semi-solid, and soft food practices before six months of age was reported from more than half of the sample. EBF was evident in 20.8% of infants, while the remainder were either SBF or weaned before six months of age (Table 1).

### 3.2. Associations between Feeding Indicators and CFM

#### 3.2.1. Association of Continuation of Breastfeeding with CFM

Compared to infants who had not received breastmilk in the last 24 h, infants with CBF had lower odds of coexistence of underweight with wasting (0.52; 95% CI: 0.31 to 0.87), underweight with stunting (0.50; 95% CI: 0.31 to 0.83), and underweight with both wasting and stunting (0.47 95% CI: 0.26 to 0.85) after adjustment for covariates. However, no association was observed between continuation of BF with coexistence of stunting with overweight/obesity (Table 2).

#### 3.2.2. Association of Predominant Feeding with CFM

Predominant feeding in infants aged below six months was not associated with any form of CFM (Table 3).

#### 3.2.3. Association of Solid, Semi-Solid, and Soft Foods with CFM

Introduction of solid, semi-solid, and soft foods during the first six months of life lowered the odds of coexistence of underweight with stunting to 0.66 (0.51 to 0.86) after adjusting for the sex of the child. However, no associations were found between the early introduction of solid, semi-solid, and soft foods and other forms of CFM (Table 4).

### 3.3. Associations between Feeding Practices and Coexisting Forms of Malnutrition

Multivariable analysis of the datasets showed around two-folds higher odds (1.96; 95% CI: 1.12 to 3.47) of coexistence of underweight with wasting among weaned infants compared to EBF infants after adjustment for covariates. Similarly, weaned infants had more than twice the odds (2.25; 95% CI: 1.16 to 4.36) of coexistence of underweight with both wasting and stunting compared with EBF infants. Conversely, there were lower odds of coexistence of stunting with overweight/obesity (0.71, 95% CI: 0.51 to 0.97) among SBF infants compared with EBF infants (Table 5). Furthermore, the relationship between different forms of SBF and CFM can be accessed from the Supplementary Table S4.

## 4. Discussion

This is the first study to examine the benefits of continuation of BF and EBF among infants aged below six months for protection against various types of CFM. In this study, the relationship of various types of CFM with feeding indicators (continuation of breastmilk, PF, and SSF) and feeding practices (EBF, SBF, and early initiation of weaning) among infants aged below six months was presented in detail. Ten different national and regional datasets were used to examine CFM among infants aged between 0 and 5.9 months. Altogether, we found malnutrition in over one-third of infants, of which half had CFM.

We found that over 85% of infants in Pakistan continued to receive maternal breastmilk until six months of age. However, less than a quarter of infants were exclusively breastfed at six months of age. At this stage of development, the National Nutritional Surveys (NNSs) of Pakistan (conducted by UNICEF) have reported EBF rates ranging from 38% in the 2011 NNS to 50% in 2001 [35]. Similarly, the PDHS reported EBF rates of 25% in 1990–1991, followed by 37% in 2006–2007, 38% in 2012–2013, and 48% in 2017–2018, respectively [26,27]. Currently, Pakistan has an EBF rate of 48%, which is close to but under the global target of 50% defined by the World Health Assembly [18]. Furthermore, this study reported that the recommended practice of EBF has been substituted by SBF (65.4%) and complete weaning practices (13.7%), which has been highlighted by other studies as pivotal barriers for effective EBF adherence during the first six months of life [36].

Findings indicate a protective role of continuation of breastfeeding for coexisting forms of undernutrition: coexistence of underweight with wasting, coexistence of underweight with stunting, and coexistence of underweight with both wasting and stunting. Similarly, this study reported two-to-three-fold higher odds of coexistence of both underweight with wasting and coexistence of underweight with wasting and stunting among infants non-adherent to EBF (completely weaned infants). Similarly, studies conducted in Denmark, Indonesia, and Pakistan also found that complete weaning increased the risk of malnutrition among infants [35,37,38,39]. On the other hand, no association of the coexistence of stunting with overweight/obesity with any feeding indicator, including continuation of breastfeeding, was seen in this study. Conversely, studies conducted in Bangladesh and Indonesia reported significantly lowered odds of coexistence of stunting with overweight/obesity among breastfed children [40]. Other studies, including a systematic review and meta-analysis found lower likelihood of both undernutrition as well as overnutrition for breastfed infants [41,42]. The lack of association between breastfeeding and coexistence of stunting with overweight/obesity in this study might be affected by SBF, because the practice of SBF in infants reduces the risk of coexistence of stunting with overweight/obesity by 0.71 (0.51 to 0.97). A study by Shaili, et al. (2014) reported a significant relationship between food quality and food quantity and infant nutritional status, rather than with type of feeding practices [43].

Infants of the richer/richest socioeconomic strata are more vulnerable to CFM compared to infants of the poorer/poorest socioeconomic strata. Our study found 1.70- (1.06 to 2.72) and 1.88-fold (1.29 to 2.74) higher odds of coexistence of underweight with wasting, and coexistence of stunting with overweight/obesity in infants of the wealthiest (high) socioeconomic status, compared to infants of the lowest socioeconomic status. In a prospective cohort study by Wijlaars et al. (2011), it was also reported that infants of low socioeconomic status at three months of age showed a significant increase in weight and height compared to infants of high socioeconomic status [44]. This relationship may change depending on the age of the child, as a recent study found that an improvement in socioeconomic status protects infants and children below five years of age from CFM [14]. Similarly, many previous studies have found that an improvement in socioeconomic status prevents various types of nutritional disorders in children, including CFM [17,45,46,47]. Further research is needed on whether there could be differences across the age of the child.

## 5. Study Strength and Limitations

To the best of our knowledge, this is the first study to examine the relationship of infant feeding practices (EBF, SBF, and early initiation of weaning) with CFM among infants aged below six months. This study analyzed ten different national and regional datasets for Pakistan, and these datasets contained data from over 10,000 children. Despite the large sample size, cross-sectional study design limits affect our findings. Temporal relationships between the CFM and different feeding practices of infants aged below six months could not be assessed. Further, information related to feeding indicators and feeding practices solely relied on verbal responses of the participants. A food list was used for collecting data pertaining to infant feeding indicators and practices, and this food list for data collection response may have compromised the validity and reliability of responses, specifically in terms of recall bias. Moreover, these surveys did not collect data related to food quantity, thus preventing us from measuring the association of CFM with total caloric intake.

## 6. Conclusions

Pediatric malnutrition is a chronic issue in Pakistan that affects more than a third of infants aged below six months. Among malnourished infants, around half are susceptible to various forms of CFM. More than two-thirds of mothers breastfeed their infants, but less than a quarter practiced EBF. Breastmilk continuation protected infants from various forms of CFM, while early initiation of weaning significantly increased the risk of coexistence of underweight with wasting and coexistence of underweight with both wasting and stunting. In contrast, the practice of SBF showed no association with any forms of CFM except coexistence of stunting with overweight/obesity. Altogether, this study found that continuation of maternal breastmilk during the first six months of life protects infants from various forms of malnutrition, including CFM. Strict policies against formula milk marketing, sales, and prescribing can prevent augmented cases of SBF and early weaning before six months, and protect infants from various types of malnutrition, including CFM.

## Figures and Tables

**Table 1:** Demographic, health, and feeding profile of children aged 0 to 5.9 months.

Variable	Category	Frequency (%) (*n*= 17,782)
**Demographic profile**		
Child age in months		2.59 ± 1.65 months
Sex	Male	8981 (50.5%)
	Female	8801 (49.5%)
History of illness in past 14 days	No	11,810 (66.4%)
	Yes	5972 (33.6%)
Maternal education	No education	9069 (51.1%)
	Primary	3155 (17.8%)
	Secondary or higher	5225 (31.1%)
Wealth index	Poorest	4066 (22.9%)
	Poorer	3823 (21.5%)
	Middle	3717 (20.9%)
	Richer	3337 (18.8%)
	Richest	2839 (16.0%)
Type of place of residence	Rural	12,088 (67.9%)
	Urban	5694 (32.1%)
**Nutritional profile**		
Total population	Healthy children	11,651 (65.5%)
	Malnourished children	6131 (34.5%)
	Standalone forms of malnutrition	3389 (19.1%)
	Coexisting forms of malnutrition	2742 (15.4%)
Standalone forms of malnutrition (55.3%, n = 3389) *	Wasting ^∞^	1594 (47%)
	Stunting ^∞^	1083 (32%)
	Underweight ^∞^	374 (11%)
	Overweight/obesity ^∞^	338 (10%)
Coexisting forms of malnutrition (44.7%, n = 2742) *	Coexistence of underweight with wasting ^¥^	846 (30.9%)
	Coexistence of underweight with stunting ^¥^	1125 (41.1%)
	Coexistence of underweight with wasting and stunting ^¥^	368 (13.4%)
	Coexistence of stunting with overweight/obesity ^¥^	403 (14.6%)
**Feeding profile**		
**Feeding indicators**		
Continuation of breastfeeding practices	No	2440 (13.7%)
	Yes	15,342 (86.3%)
Predominant feeding (PF) practices	No	9693 (54.5%)
	Yes	8089 (45.5%)
Solid and semisolid food (SSF) practices	No	6716 (37.8%)
	Yes	11,066 (62.2%)
**Feeding practices**		
Derived feeding practices	Exclusive breastfeeding (EBF)	3708 (20.8%)
	Supplementary breastfeeding (SBF)	11,637 (65.4%)
	Weaning	2441 (13.7%)

* = Denominator for calculating standalone and coexisting forms of malnutrition was the prevalence of malnourished children in Pakistan (n = 6131). ∞ = Denominator for calculating wasting, stunting, underweight, and overweight/obesity was the prevalence of standalone forms of malnutrition (n = 3389). ¥ = Denominator for calculating coexistence of underweight with wasting, coexistence of underweight with stunting, coexistence of underweight with both wasting and stunting, and coexistence of stunting with overweight/obesity was the prevalence of coexisting forms of malnutrition (n = 2742).

**Table 2:** Multinomial adjusted model for the associations between continuation of breastfeeding and CFM.

Variable	Categories	Coexistence of Underweight with Wasting ^1^	Coexistence of Underweight with Stunting ^2^	Coexistence of Underweight with Wasting and Stunting ^3^	Coexistence of Stunting with Overweight/Obesity ^4^
**Continuation of breastfeeding (CBF) practices**	No	Ref	Ref	Ref	Ref
	Yes	0.52 (0.31 to 0.87) *	0.50 (0.31 to 0.83) *	0.47 (0.26 to 0.85) *	0.97 (0.75 to 1.24)
**Age**					0.97 (0.75 to 1.24)
**Sex**	Male		Ref	Ref	-
	Female		0.67 (0.53 to 0.85) *	0.72 (0.53 to 0.96) *	
**Health status**	No		-	-	Ref
	Yes				0.71 (0.55 to 0.91) *
**Maternal education**	No education			Ref	-
	Primary			0.67 (0.45 to 1.01)	
	Secondary or Higher			0.60 (0.40 to 0.89) *	
**Socioeconomic status**	Poorest	Ref		-	Ref
	Poorer	0.95 (0.68 to 1.32)			1.04 (0.74 to 1.45)
	Middle	1.12 (0.78 to 1.57)			1.15 (0.81 to 1.66)
	Richer	1.72 (1.48 to 2.60) *			1.44 (0.99 to 2.10)
	Richest	1.70 (1.06 to 2.71) *			1.82 (1.25 to 2.64) *
**Type of place of residence**	Rural	-		Ref	-
	Urban			1.58 (1.12 to 2.23) *	

* = Significant association of outcome variable either with outcome and/or covariates (*p* < 0.05). 1 = Adjusted for exclusive breastfeeding practices with socioeconomic status. 2 = Adjusted for exclusive breastfeeding practices with child sex. 3 = Adjusted for exclusive breastfeeding practices with child sex, maternal education, and type of place of residence. 4 = Adjusted for exclusive breastfeeding practices with child age, health status, and socioeconomic status.

**Table 3:** Multinomial adjusted model for the associations between predominant feeding and CFM.

Variable	Categories	Coexistence of Underweight with Wasting ^1^	Coexistence of Underweight with Stunting ^2^	Coexistence of Underweight with Wasting and Stunting ^3^	Coexistence of Stunting with Overweight/Obesity ^4^
**Predominant feeding (PF) practices**	No	Ref	Ref	Ref	Ref
	Yes	1.09 (0.85 to 1.40)	0.87 (0.69 to 1.11)	1.12 (0.83 to 1.51)	0.81 (0.62 to 1.03)
**Age**					0.89 (0.82 to 0.97) *
**Sex**	Male		Ref	Ref	-
	Female		0.68 (0.54 to 0.86) *	0.73 (0.54 to 0.98) *	
**Health status**	No		-	-	Ref
	Yes				0.70 (0.55 to 0.90) *
**Maternal education**	No education			Ref	-
	Primary			0.66 (0.44 to 0.99) *	
	Secondary or Higher			0.62 (0.41 to 0.92) *	
**Socioeconomic status**	Poorest	Ref		-	Ref
	Poorer	0.96 (0.69 to 1.34)			1.05 (0.75 to 1.46)
	Middle	1.15 (0.81 to 1.63)			1.17 (0.81 to 1.67)
	Richer	1.79 (1.19 to 2.71) *			1.46 (1.01 to 2.12) *
	Richest	1.82 (1.14 to 2.90) *			1.81 (1.25 to 2.62) *
**Type of place of residence**	Rural	-		Ref	-
	Urban			1.51 (1.07 to 2.13) *	

* = Significant association of outcome variable either with outcome and/or covariates (*p* < 0.05). 1 = Adjusted for predominant feeding practices with socioeconomic status. 2 = Adjusted for predominant feeding practices with child sex. 3 = Adjusted for predominant feeding practices with child sex, maternal education, and type of place of residence. 4 = Adjusted for predominant feeding practices with child age, health status, and socioeconomic status.

**Table 4:** Multinomial adjusted model for associations between use of solid, semi-solid, and soft foods and CFM.

Variable	Categories	Coexistence of Underweight with Wasting ^1^	Coexistence of Underweight with Stunting ^2^	Coexistence of Underweight with Wasting and Stunting ^3^	Coexistence of Stunting with Overweight/Obesity ^4^
**Solid and semisolid food (SSF) practices**	No	Ref	Ref	Ref	Ref
	Yes	1.05 (0.80 to 1.40)	0.66 (0.51 to 0.86) *	1.03 (0.74 to 1.44)	1.04 (0.81 to 1.32)
**Age**					0.87 (0.81 to 0.94) *
**Sex**	Male		Ref	Ref	-
	Female		0.67 (0.53 to 0.85) *	0.73 (0.54 to 0.98) *	
**Health status**	No			-	Ref
	Yes				0.71 (0.55 to 0.91) *
**Maternal education**	No education			Ref	-
	Primary			0.65 (0.44 to 0.97) *	
	Secondary or Higher			0.61 (0.41 to 0.91) *	
**Socioeconomic status**	Poorest	Ref		-	Ref
	Poorer	0.97 (0.69 to 1.35)			1.04 (0.74 to 1.45)
	Middle	1.15 (0.81 to 1.62)			1.15 (0.80 to 1.65)
	Richer	1.78 (1.18 to 2.68) *			1.44 (0.99 to 2.09)
	Richest	1.80 (1.13 to 2.87) *			1.82 (1.25 to 2.64) *
**Type of place of residence**	Rural	-		Ref	-
	Urban			1.52 (1.08 to 2.15) *	

* = Significant association of outcome variable either with outcome and/or covariates (*p* < 0.05). 1 = Adjusted for solid and semi-solid food feeding practices with socioeconomic status. 2 = Adjusted for solid and semi-solid food feeding practices with child sex. 3 = Adjusted for solid and semi-solid food feeding practices with child sex, maternal education, and type of place of residence. 4 = Adjusted for solid and semi-solid food feeding practices with child age, health status, and socioeconomic status.

**Table 5:** Multinomial adjusted model for assessing the determinants of coexisting forms of malnutrition.

Variable	Categories	Coexistence of Underweight with Wasting ^1^	Coexistence of Underweight with Stunting ^2^	Coexistence of Underweight with Wasting and Stunting ^3^	Coexistence of Stunting with Overweight/Obesity ^4^
**Feeding practices**	Exclusive breastfeeding (EBF)	Ref	Ref	Ref	Ref
	Supplementary breastfeeding (SBF)	1.10 (0.81 to 1.51)	0.84 (0.63 to 1.14)	1.16 (0.78 to 1.71)	0.71 (0.51 to 0.97) *
	Early initiation of weaning	1.96 (1.12 to 3.47) *	1.65 (0.95 to 2.85)	2.25 (1.16 to 4.36) *	0.81 (0.58 to 1.12)
**Age**		-			0.89 (0.82 to 0.96) *
**Sex**	Male		Ref	Ref	-
	Female		0.67 (0.53 to 0.85) *	0.72 (0.53 to 0.97) *	
**Health status**	No		-	-	Ref
	Yes				0.71 (0.55 to 0.90) *
**Maternal education**	No education			Ref	-
	Primary			0.68 (0.46 to 1.02)	
	Secondary or Higher			0.61 (0.41 to 0.91) *	
**Socioeconomic status**	Poorest	Ref		-	Ref
	Poorer	0.95 (0.68 to 1.33)			1.03 (0.74 to 1.45)
	Middle	1.11 (0.78 to 1.57)			1.18 (0.82 to 1.70)
	Richer	1.73 (1.15 to 2.61) *			1.48 (1.01 to 2.15) *
	Richest	1.70 (1.06 to 2.72) *			1.88 (1.29 to 2.74) *
**Type of place of residence**	Rural	-		Ref	-
	Urban			1.58 (1.12 to 2.24) *	

* = Significant association of outcome variable either with outcome and/or covariates. 1 = Results adjusted for infant feeding practices and socioeconomic status; 2 = Results adjusted for infant feeding practices and child sex; 3 = Results adjusted for infant feeding practices and child sex, maternal education, and type of place of residence; 4 = Results adjusted for infant feeding practices and child age, health status, and socioeconomic status.

## Data Availability

The data from this study can be retrieved from the DHS program (www.dhsprogram.com) and UNICEF (www.mics.unicef.org/surveys).

## Supplementary Materials

The following supporting information can be downloaded at: https://www.mdpi.com/article/10.3390/nu14204242/s1, Supplementary Table S1: Described the process of data screening, data cleaning, and data transformation using PDHS and MICS datasets. Supplementary Table S2: Showed different infant feeding variables and their use for defining various types of infant feeding indicators, infant feeding practices and four different types of SBF. Supplementary Table S3: Presented the unadjusted odds for assessing the determinants of various forms of coexisting forms of malnutrition. Supplementary Table S4: Measured the association of each form of Supplementary breastfeeding with the various forms of coexisting forms of malnutrition.
