# Supplementary material for: Association of Infant Feeding Indicators and Infant Feeding Practices with Coexisting Forms of Malnutrition in Children under Six Months of Age
Source: Nutrients. 2022 Oct 12;14(20):4242. doi: 10.3390/nu14204242 (PMC9608454; doi:10.3390/nu14204242)
Supplement: Supplementary file 1 [file nutrients-14-04242-s001.zip › nutrients-1927635-supplementary.pdf]

Infant Feeding Practices and Coexisting Forms of Malnutrition in children under six months of age

Supplementary file 1

Table S1: Data screening, cleaning, and data transformation process of DHS and MICS datasets

Survey name	MICS-6		MICS-5				MICS-4		PDHS		Total
**Region covered**	**Sindh**	**Punjab**	**Punjab**	**Sindh**	**KPK**	**GB**	**Punjab**	**Baluchistan**	**2012-2013**	**2017-2018**	**-**
**Year**	2018-2019	2017-2018	2014	2014	2016-2017	2016-2017	2011	2010	2012-2013	2017-2018	-
**Total sample size**	18,312	42,408	31,083	18,108	21,359	7,005	74,126	10,432	11,763	12,708	247,304
**Exclude: Missing age**	(335)	(2,609)	(3,588)	(1,503)	(433)	(368)	(7,460)	(698)	(7,806)	(8,209)	(33,009)
**Children aged 0 to 59.9 mo.**	17,977	39,799	27,495	16,605	20,926	6,637	66,666	9,734	3,957	4,499	214,295
**Exclude: Children 6 to 59.9 mo**	(15,132)	(33,409)	(24,243)	(14,360)	(17,486)	(5,934)	(58,378)	(7,778)	(3,319)	(3,874)	(183,913)
**Children aged 0 to 5.9 mo**	2,845	6,390	3,252	2,245	3,440	703	8,288	1,956	638	625	30,382
**Exclude: Missing anthropometry including refusals**	(90)	(503)	(188)	(191)	(298)	(23)	(797)	(1,072)	(110)	(107)	(3,379)
**Children with complete anthropometry**	2,755	5,887	3,064	2,054	3,142	680	7,491	884	528	518	27,003
**Exclude: Anthropometric outliers**	(639)	(744)	(260)	(263)	(699)	(46)	(827)	(652)	(254)	(102)	(4,486)
**Children with appropriate anthropometry**	2,116	5,143	2,804	1,791	2,443	634	6,664	232	274	416	22,517
**Exclude: Missing dietary information**	(683)	(1,288)	(696)	(344)	(477)	(65)	(1,113)	(28)	(9)	(12)	(4,715)
**Data of children aged 0-5.9 months for analysis**	1,413	3,855	2108	1,447	1,966	569	5,551	204	265	404	17,782

Table S2-A: Variables for Infant feeding practices

Variable	Question	Categories with coding
V001	Did the child drink Breastmilk in the last 24 hours?	Yes = 1<br>No = 0
V002	Did the child drink ORS in the last 24 hours?	Yes = 1<br>No = 0
V003	Did the child drink multivitamins including vitamin A in the last 24 hours?	Yes = 1<br>No = 0
V004	Did the child drink iron & folate in the last 24 hours?	Yes = 1<br>No = 0
V005	Did the child drink water in the last 24 hours?	Yes = 1<br>No = 0
V006	Did the child drink clear juice in the last 24 hours?	Yes = 1<br>No = 0
V007	Did the child drink clear broth in the last 24 hours?	Yes = 1<br>No = 0
V008	Did the child drink tea without milk in the last 24 hours?	Yes = 1<br>No = 0
V009	Did the child drink animal milk in the last 24 hours?	Yes = 1<br>No = 0
V010	Did the child drink formula milk in the last 24 hours?	Yes = 1<br>No = 0
V011	Did the child drink/eat yogurt in the last 24 hours?	Yes = 1<br>No = 0
V012	Did the child eat any solid or semi-solid or soft food or liquid (other than milk) in the last 24 hours?	Yes = 1<br>No = 0

Table S2-B: Variable selections for defining each feeding indicator

Feeding indicator	Variable selected
Continuation of breastfeeding (BF)	V001-Did the child drink Breastmilk in the last 24 hours?
Predominant feeding (PF)	V002-Did the child drink ORS in the last 24 hours?
	V003-Did the child drink multivitamins including vitamin A in the last 24 hours?
	V004-Did the child drink iron & folate in the last 24 hours?
	V005-Did the child drink water in the last 24 hours?
	V006-Did the child drink clear juice in the last 24 hours?
	V007-Did the child drink clear broth in the last 24 hours?
	V008-Did the child drink tea without milk in the last 24 hours?
Solid or semi-solid or soft food feeding (SSF)	V009-Did the child drink animal milk in the last 24 hours?
	V010-Did the child drink formula milk in the last 24 hours?
	V011-Did the child drink/eat yogurt in the last 24 hours?
	V012-Did the child eat any solid or semi-solid or soft food or liquid (other than milk) in the last 24 hours?

Table S2-C: Variable selections for defining each feeding practice

Feeding indicator	Variable selected			
	V001	V002, V003, V004	V005, V006, V007, V008	V009, V010, V011, V012
Exclusive breastfeeding (EBF)	Yes	Either Yes or No	No	No
Supplemental feeding (SBF)	Yes		Yes for at least one variable	
Early weaning practices	No			

Table S2-D: Variable selections for defining each type of supplementary breastfeeding practice

Feeding indicator	Variable selected					
	V001	V002, V003, V004	V005, V006, V007, V008	V009	V010	V011, V012
Breastmilk with infant formula	Yes	Either Yes or No		Yes		
Breastmilk with animal milk					Yes	
Breastmilk with water, juice, broth, and other liquid			Yes for at least one variable			

Breastmilk with solid, semi-solid and soft foods									Yes for at least one variable

Supplementary file 3

Table S3: Unadjusted odds for assessing the determinants of Coexisting forms of malnutrition

**Variable**	**Categories**	**Coexistence of underweight with wasting**	**Coexistence of underweight with stunting**	**Coexistence of underweight with wasting and stunting**	**Coexistence of stunting with overweight/obesity**
**Exclusive breastfed (EBF) practices**	No	Ref	Ref	Ref	Ref
	Yes	0.47 (0.28 to 0.80) *	0.51 (0.31 to 0.84) *	0.51 (0.28 to 0.91) *	0.90 (0.71 to 1.14)
**Predominant feeding (PF) practices**	No	Ref	Ref	Ref	Ref
	Yes	1.05 (0.82 to 1.34)	0.86 (0.68 to 1.10)	1.22 (0.91 to 1.62)	0.72 (0.57 to 0.92) *
**Solid, & semisolid, food (SSF) practices**	No	Ref	Ref	Ref	Ref
	Yes	1.10 (03.83 to 1.45)	0.68 (0.52 to 0.88) *	1.01 (0.73 to 1.41)	1.02 (0.81 to 1.29)
**Early weaning practices[1]**	Exclusive breastfeeding (EBF)	Ref	Ref	Ref	Ref
	Partially weaned	1.11 (0.81 to 1.52)	0.85 (0.63 to 1.15)	1.17 (0.81 to 1.72)	0.71 (0.52 to 0.96) *
	Completely weaned	2.15 (1.22 to 3.78) *	1.65 (0.95 to 2.84)	2.12 (1.11 to 4.05) *	0.87 (0.63 to 1.19)
**Age**		0.99 (0.91 to 1.07)	1.06 (0.97 to 1.15)	1.04 (0.94 to 1.15)	0.87 (0.81 to 0.94) *
**Sex**	Male	Ref	Ref	Ref	Ref
	Female	0.83 (0.65 to 1.06)	0.68 (0.53 to 0.86) *	0.73 (0.55 to 0.98) *	0.94 (0.74 to 1.18)
**Health status**	No	Ref	Ref	Ref	Ref
	Yes	0.81 (0.63 to 1.05)	0.94 (0.74 to 1.20)	0.88 (0.64 to 1.16)	0.67 (0.53 to 0.86) *
**Maternal education**	No education	Ref	Ref	Ref	Ref
	Primary	1.06 (0.77 to 1.45)	0.80 (0.58 to 1.09)	0.67 (0.45 to 0.99) *	0.98 (0.70 to 1.38)
	Secondary or Hig her	1.26 (0.92 to 1.71)	0.99 (0.73 to 1.34)	0.69 (0.47 to 1.01)	
**Socioeconomic status**	Poorest	Ref	Ref	Ref	

	Poorer	0.97 (0.69 to 1.35)	0.87 (0.64 to 1.19)	0.72 (0.49 to 1.07)	
	Middle	1.15 (0.81 to 1.63)	0.86 (0.62 to 1.20)	0.68 (0.45 to 1.04)	
	Richer	1.79 (1.19 to 2.70) *	1.18 (0.79 to 1.77)	1.18 (0.73 to 1.89)	
	Richest	1.81 (1.13 to 2.88) *	1.24 (0.79 to 1.96)	1.01 (0.58 to 1.75)	
**Type of place of residence**	Rural	Ref	Ref	Ref	
	Urban	1.39 (1.05 to 1.84) *	1.28 (0.97 to 1.68)	1.42 (1.02 to 1.98) *	

Supplementary file 4

Table S4-A: Assessing the association of various types of SBF with the coexistence of underweight with wasting (Underweight as reference)

Variable	Categories	Unadjusted Odds (95% CI)	Adjusted Odds[1] (95% CI)	Adjusted Odds[2] (95% CI)	Adjusted Odds[3] (95% CI)	Adjusted Odds[4] (95% CI)
**Coadministration of BF with formula milk**	EBF	Ref	Ref			
	SBF-1	1.02 (0.64 to 1.62)	1.04 (0.64 to 1.68)			
**Coadministration of BF with animal milk**	EBF	Ref		Ref		
	SBF-2	1.12 (0.85 to 1.46)		1.02 (0.77 to 1.35)		
**Coadministration of BF with water, juice & other liquids**	EBF	Ref			Ref	
	SBF-3	1.23 (0.58 to 2.57)			0.82 (0.38 to 1.78)	
**Coadministration of BF with solid, semi-solid, and soft foods**	EBF	Ref				Ref
	SBF-4	0.77 (0.53 to 1.10)				0.82 (0.57 to 1.20)
**Age**		0.99 (0.91 to 1.08)				
**Sex**	Male	Ref				
	Female	0.85 (0.65 to 1.07)				
**Health status**	No	Ref				
	Yes	0.84 (0.63 to 1.13)				
**Maternal education**	No education	Ref				
	Primary	1.10 (0.82 to 1.46)				
	Secondary or Higher	1.24 (0.87 to 1.77)				
**Socioeconomic status**	Poorest	Ref				
	Poorer	0.95 (0.69 to 1.33)				
	Middle	1.11 (0.78 to 1.57)				

	Richer	1.76 (1.17 to 2.66) *				
	Richest	1.78 (1.11 to 2.83) *				
**Type of place of residence**	Rural	Ref	Ref	Ref	Ref	Ref
	Urban	0.66 (0.45 to 0.96) *	2.10 (1.42 to 3.11) *	2.10 (1.41 to 3.11) *	0.47 (0.32 to 0.71) *	2.07 (1.40 to 3.07) *

1 = Adjusted for coadministration of breastfeeding & infant formula with the type of place of residence.

2 = Adjusted for coadministration of breastfeeding & animal milk with the type of place of residence.

3 = Adjusted for coadministration of breastfeeding & water, juice & other liquids with the type of place of residence.

4 = Adjusted for coadministration of breastfeeding & solid, semi-solid & soft food with the type of place of residence.

Table S4-B: Assessing the association of various types of SBF with the coexistence of underweight with stunting (Underweight as reference)

Variable	Categories	Unadjusted Odds (95% CI)	Adjusted Odds[1] (95% CI)	Adjusted Odds[2] (95% CI)	Adjusted Odds[3] (95% CI)	Adjusted Odds[4] (95% CI)
**Coadministration of BF with formula milk**	EBF	Ref	Ref			
	SBF-1	0.94 (0.60 to 1.48)	0.94 (0.59 to 1.50)			
**Coadministration of BF with animal milk**	EBF	Ref		Ref		
	SBF-2	0.85 (0.65 to 1.11)		0.84 (0.65 to 1.11)		
**Coadministration of BF with water, juice & other liquids**	EBF	Ref			Ref	
	SBF-3	1.02 (0.49 to 2.11)			1.12 (0.52 to 2.38)	
**Coadministration of BF with solid, semi-solid, and soft foods**	EBF	Ref				Ref
	SBF-4	0.95 (0.68 to 1.34)				0.87 (0.62 to 1.24)
**Age**		0.93 (0.86 to 1.01)				1.09 (1.00 to 1.19) *
**Sex**	Male	Ref	Ref	Ref	Ref	Ref
	Female	0.68 (0.53 to 0.86) *	0.67 (0.53 to 0.86) *	0.68 (0.53 to 0.86) *	0.68 (0.53 to 0.86) *	0.68 (0.53 to 0.86) *
**Health status**	No	Ref				Ref
	Yes	0.77 (0.58 to 1.02)				0.74 (0.56 to 0.99) *
**Maternal education**	No education	Ref				
	Primary	0.85 (0.64 to 1.12)				
	Secondary or Higher	0.99 (0.70 to 1.40)				
**Socioeconomic status**	Poorest	Ref				
	Poorer	0.87 (0.64 to 1.19)				
	Middle	0.85 (0.61 to 1.18)				

	Richer	1.16 (0.78 to 1.74)				
	Richest	1.24 (0.79 to 1.96)				
**Type of place of residence**	Rural	Ref				
	Urban	1.32 (0.99 to 1.75)				

1 = Adjusted for coadministration of breastfeeding & infant formula with the child sex.

2 = Adjusted for coadministration of breastfeeding & animal milk with the child sex.

3 = Adjusted for coadministration of breastfeeding & water, juice & other liquids with the child sex

4 = Adjusted for coadministration of breastfeeding & solid, semi-solid & soft food with the child's age, sex, and illness.

Table S4-C: Assessing the association of various types of SBF with the coexistence of underweight with both wasting & stunting (Underweight as reference)

Variable	Categories	Unadjusted Odds (95% CI)	Adjusted Odds[1] (95% CI)	Adjusted Odds[2] (95% CI)	Adjusted Odds[3] (95% CI)	Adjusted Odds[4] (95% CI)
**Coadministration of BF with formula milk**	EBF	Ref	Ref			
	SBF-1	0.74 (0.41 to 1.35)	0.69 (0.37 to 1.30)			
**Coadministration of BF with animal milk**	EBF	Ref		Ref		
	SBF-2	1.08 (0.78 to 1.49)		1.08 (0.78 to 1.51)		
**Coadministration of BF with water, juice & other liquids**	EBF	Ref			Ref	
	SBF-3	1.51 (0.66 to 3.45)			1.42 (0.58 to 3.43)	
**Coadministration of BF with solid, semi-solid, and soft foods**	EBF	Ref				Ref
	SBF-4	0.93 (0.61 to 1.43)				0.89 (0.58 to 1.37)
**Age**		1.04 (0.94 to 1.15)				
**Sex**	Male	Ref	Ref	Ref	Ref	Ref
	Female	0.73 (0.55 to 0.98) *	0.69 (0.51 to 2.32) *	0.69 (0.51 to 0.93) *	0.69 (0.51 to 0.93) *	0.69 (0.51 to 0.93) *
**Health status**	No	Ref				
	Yes	0.89 (0.64 to 1.25)				
**Maternal education**	No education	Ref				
	Primary	0.62 (0.44 to 0.89) *	Ref	Ref	Ref	Ref
	Secondary or High er	0.80 (0.52 to 1.23)	0.60 (0.41 to 0.86) *	0.59 (0.41 to 0.86) *	0.60 (0.41 to 0.86) *	0.59 (0.41 to 0.86) *
**Socioeconomic status**	Poorest	Ref	0.68 (0.43 to 1.08)	0.67 (0.42 to 1.06)	0.67 (0.42 to 1.06)	0.66 (0.42 to 1.06)
	Poorer	0.72 (0.49 to 1.06)				

	Middle	0.67 (0.44 to 1.01)				
	Richer	1.17 (0.73 to 1.87)				
	Richest	1.00 (0.57 to 1.74)				
**Type of place of residence**	Rural	Ref	Ref	Ref	Ref	Ref
	Urban	1.48 (1.06 to 2.08) *	1.63 (1.14 to 2.32) *	1.61 (1.13 to 2.29) *	1.59 (1.11 to 2.26) *	1.61 (1.13 to 2.30) *

1 = Adjusted for coadministration of breastfeeding & infant formula with the child sex, maternal education, and type of place of residence.
2 = Adjusted for coadministration of breastfeeding & animal milk with the child sex, maternal education, and type of place of residence.
3 = Adjusted for coadministration of breastfeeding & water, juice & other liquids with the child sex, maternal education, and type of place of residence.
4 = Adjusted for coadministration of breastfeeding & solid, semi-solid & soft food with the child sex, maternal education, and type of place of residence.

Table S4-D: Assessing the association of various types of SBF with the coexistence of Stunting with Overweight/Obesity (Stunting as reference)

**Variable**	**Categories**	**Unadjusted Odds (95% CI)**	**Adjusted Odds[1] (95% CI)**	**Adjusted Odds[2] (95% CI)**	**Adjusted Odds[3] (95% CI)**	**Adjusted Odds[4] (95% CI)**
**Coadministration of BF with formula milk**	EBF	Ref	Ref			
	SBF-1	1.20 (0.76 to 1.91)	1.12 (0.69 to 1.81)			
**Coadministration of BF with animal milk**	EBF	Ref		Ref		
	SBF-2	0.69 (0.52 to 0.92) *		0.76 (0.57 to 1.03)		
**Coadministration of BF with water, juice & other liquids**	EBF	Ref			Ref	
	SBF-3	0.82 (0.40 to 1.67)			0.96 (0.46 to 2.01)	
**Coadministration of BF with solid, semi-solid, and soft foods**	EBF	Ref				Ref
	SBF-4	0.82 (0.56 to 1.20)				0.93 (0.62 to 1.40)
**Age**		0.85 (0.79 to 0.92) *	0.86 (0.79 to 0.93) *	0.86 (0.80 to 0.93) *	0.85 (0.79 to 0.93) *	0.86 (0.79 to 0.93) *
**Sex**	Male	Ref				
	Female	0.97 (0.77 to 1.23)				
**Health status**	No	Ref	Ref	Ref	Ref	Ref
	Yes	0.59 (0.43 to 0.81) *	0.61 (0.43 to 0.84) *	0.61 (0.44 to 0.85) *	0.61 (0.44 to 0.84) *	0.61 (0.44 to 0.84) *
**Maternal education**	No education	Ref				
	Primary	1.14 (0.85 to 1.53)				
	Secondary or Higher	1.57 (1.17 to 2.11)				
**Socioeconomic status**	Poorest	Ref	Ref	Ref	Ref	Ref
	Poorer	1.08 (0.77 to 1.53)	1.12 (0.79 to 1.58)	0.79 (0.54 to 1.16)	0.80 (0.55 to 1.17)	0.80 (0.55 to 1.16)

	Middle	1.17 (0.81 to 1.69)	1.23 (0.85 to 1.80)	0.89 (0.61 to 1.28)	0.90 (0.62 to 1.31)	0.90 (0.62 to 1.30)
	Richer	1.44 (0.99 to 2.12)	1.47 (1.00 to 2.18) *	1.19 (0.79 to 1.80)	1.19 (0.79 to 1.80)	1.19 (0.79 to 1.79)
	Richest	1.75 (1.20 to 2.55) *	1.68 (1.14 to 2.49) *	1.38 (0.92 to 2.07)	1.38 (0.92 to 2.07)	1.38 (0.92 to 2.07)
**Type of place of residence**	Rural	Ref				
	Urban	1.04 (0.80 to 1.35)				

1 = Adjusted for coadministration of breastfeeding & infant formula with the child's age, health status, and wealth index.

2 = Adjusted for coadministration of breastfeeding & animal milk with the child's age, health status, and wealth index.

3 = Adjusted for coadministration of breastfeeding & water, juice & other liquids with the child's age, health status, and wealth index.

4 = Adjusted for coadministration of breastfeeding & solid, semi-solid & soft food with the child's age, health status, and wealth index.
